# Vancomycin-Iridium (III) Interaction: An Unexplored Route for Enantioselective Imine Reduction

**DOI:** 10.3390/molecules24152771

**Published:** 2019-07-30

**Authors:** Giorgio Facchetti, Sara Pellegrino, Raffaella Bucci, Donatella Nava, Raffaella Gandolfi, Michael S. Christodoulou, Isabella Rimoldi

**Affiliations:** Dipartimento di Scienze Farmaceutiche, Università degli Studi di Milano, Via Venezian 21, 20133 Milano, Italy

**Keywords:** glycopeptides, hybrid catalyst, asymmetric hydrogen transfer

## Abstract

The chiral structure of antibiotic vancomycin (Van) was exploited as an innovative coordination sphere for the preparation of an IrCp* based hybrid catalysts. We found that Van is able to coordinate iridium (Ir(III)) and the complexation was demonstrated by several analytical techniques such as MALDI-TOF, UV, Circular dichroism (CD), Raman IR, and NMR. The hybrid system so obtained was employed in the Asymmetric Transfer Hydrogenation (ATH) of cyclic imines allowing to obtain a valuable 61% *e.e.* (*R*) in the asymmetric reduction of quinaldine **2**. The catalytic system exhibited a saturation kinetics with a calculated efficiency of K_cat_/K_M_ = 0.688 h^−1^mM^−1^.

Vancomycin (Van) is a front-line glycopeptide antibiotic produced by *Streptomyces orientalis* and active against Gram positive infections. Its activity is due to a selective binding to the D-Ala-D-Ala terminus of peptidoglycan precursor hampering the formation of the bacterial cell wall [1,2]. Recently, it was found that several biological effects of Van are also related to its ability to bind both Cu(II) and Zn(II) metal ions under physiological/neutral conditions [3,4,5]. Van is a macrocycle characterized by axially chiral biaryl structural motifs [6,7,8,9] connected to a glycopeptide chain endowed with different groups promoting dipole-dipole interactions and pi-pi interactions. Its copper binding site involves *N*-terminal imino nitrogen, two consecutive nitrogen atoms in the peptide chain, and one oxygen atom from asparagine amide group. This ability to form a stable complex with transition metals, the atropoisomerism induced by the restricted rotation around the aryl-aryl bonds, and the macrocyclic chiral basket-like structure all make Van an interesting, although yet unexplored, ligand for asymmetric catalysis.

In the last decades, the development of hybrid catalysts that combine the advantages of chemical catalysts and biocatalysis, has launched an original approach allowing high selectivity and specificity to be merged with a wide scope of reactivity and substrates. The widespread presence of metal ions in biological systems and the possibility of using well-known natural structures as ligands in metal complexes have inspired different research groups to exploit biodiversity and to investigate new artificial systems, such as the combination of the reactive metals with different biological scaffolds (e.g., proteins, DNA, and peptides) [10,11,12,13,14,15,16,17].

In this work, we verified the ability of Van to coordinate Ir(III) in a different manner compared to Cu(II) and Zn(II). The catalytic performance of such a complex in which Van represented the source of chirality in transition metal complex, was evaluated in the asymmetric reduction of cyclic imines. This reaction is a key tool to produce complex alkaloids and un-natural β-amino acids as well as important pharmaceutical intermediates [18,19,20,21]. In this regard, Asymmetric Transfer Hydrogenation (ATH) provides a valuable process and Ir(III) catalyzed ATH has been recently widely investigated due to the possibility to use environmentally friendly reaction conditions as the aqueous media for both the reduction of imines and ketones [22,23,24,25,26,27,28]. Generally, ruthenium and rhodium were used as co-factor for transfer hydrogenation reactions for involving ketones, even if in the last decades many researchers shed light on the potential use of iridium based catalysts, thus expanding the scope of reaction to imines. These catalytic systems afforded better results both in terms of enantioselectivity and conversion rate under aqueous conditions, desirable and green chemistry compatible ones [29,30,31,32]. Furthermore, in our group, we recently developed different catalytic artificial imine reductases in which the active moiety was represented by a η^5^-piano stool Ir(III) complex [33,34].

Van ability to coordinate an Ir(III) center was assessed in solution using dimeric [IrCp*Cl_2_]_2_ in a 0.5:1 ratio with Van. The formation of the complex was confirmed by several analytical techniques such as MALDI-TOF, UV, Circular dichroism (CD), Raman IR, and NMR. First, MALDI-TOF experiments showed the presence of the molecular peak corresponding to a monomer complex, while no peaks corresponding to alternative complexes were observed (see SI, Figure S1). The coordination of Van to the iridium metal center was then confirmed by the bathochromic shift of the metal-to-ligand charge transfer transition (MLCT) band [35] in the UV spectra (Ɛ_λ436nm_ = 6 × 10^3^ M^−1^cm^−1^) [36,37]. Interestingly, the addition of [IrCp*Cl_2_]_2_ to a Van solution caused a shift of the maximum absorbance from 417 nm for [IrCp*Cl_2_]_2_ alone to 429 nm for the [Ir(Cp*)(Van)Cl] complex [38,39] (see SI, Figure S2). Circular dichroism (CD) experiments on free vancomycin and on the Ir/Van 1:1 complex further confirmed that the complexation between the chiral macromolecule and the metal was effective.

In Figure 1, the CD spectra in acetate buffer (pH 5, on the left) and in phosphate buffer (pH 8, on the right) are reported. By comparing free Van spectra (black lines) and the Van/Ir 1:1 complex spectra (red lines), the appearance of negative Cotton effects at around 360 nm together with slightly positive shoulders at around 415 nm was observed both at acidic and basic pH. The binding with the metal is particularly evident by comparing the spectra of the free Van (black lines) with the ones obtained by subtracting Van contribution from the 1:1 mixture spectra. The resulting curves (blue lines) showed the induction of chirality at the metal center, as the observed Cotton effects could be ascribable only to iridium energetic transitions. Furthermore, looking at the 1:1 complex spectra, a different behavior of Van at 285 nm band is observed depending on pH. In acetate buffer, an increase in the intensity was observed while the intensity of the same band decreases in phosphate buffer. From these findings we can assume that Van aromatic rings are involved in the coordination, but assume a different conformation depending on the environment.

NMR experiments were then performed to shed more light on the complexation mode. From both ^1^H and ^13^C NMR spectra, it was established that the iridium coordination affected both the sugar and the aromatic parts of Van (Figure 2 and SI), suggesting a conformational rearrangement of the molecule upon binding. The effect on the aromatic rings and on the CH_3_ groups was further confirmed by RAMAN spectroscopy [40,41] (see SI, Figure S5).

The coordination mode between the chemical groups involved with Van and the metal ion, however, remains uncertain in the absence of crystals suitable for X-ray structural assignment. This unclear coordination mode could depend on the different Lewis bases present in the Van: the two adjacent but not consecutive nitrogens of the amide and -NHCH_3_ group, the carboxylate on the C-terminus, the 2,2-biphenolic units, and the oxygen atom of the Asn sidechain. All these ones could be a possible coordination site between the Van and the iridium metal center, in function of the pH and of the ionic strength.

The [Ir(Cp*)(Van)Cl] complex was prepared in situ after 1 h pre-complexation time and its catalytic activity was evaluated in the asymmetric transfer hydrogenation reactions of three cyclic imines **A**, **B** and **C**, having different electronic and steric properties, and chosen for being precursors of pharmaceutically valuable intermediates [42] (Scheme 1).

Different reaction conditions were screened: buffer, pH, temperature, substrate concentration, substrate/catalyst ratio, Van/Ir pre-catalyst ratio, and (see Table T2 in SI) [43,44,45,46] different pre-complexation times were also evaluated by ESI-MS (spectra not reported).

The optimized reaction conditions were set up as reported in Table 1: pre-complexation time (1 h), substrate/catalyst ratio of 50:1, final substrate concentration (16 mM), Van/Ir pre-catalyst ratio 2:1, HCOONa 3 M as hydrogen donor at room temperature.

In the reduction of 6,7-dimethoxy-1-methyl-3,4-dihydroisoquinoline **1**, an appreciable conversion was obtained (up to 82%, Table 1, Entry 3, Sub **1**) although an enantioselectivity of 4%. The *e.e.* was increased to 20% for the *R* enantiomer when the reaction was conducted in a phosphate buffer pH 8 (Table 1, Entry 1, Sub **1**).

The best result in terms of enantioselectivity in the ATH of quinaldine **2** was achieved by performing the reaction in a MES 1.2 M buffer at pH 5 with a significant 61% *e.e.* despite a modest 35% conversion rate (Table 1, Entry 6, Sub **2**). Interestingly, an inversion of configuration was observed in the ATH of 3-methylbenzo[d]isothiazole 1,1-dioxide **3** going from basic pH (42% *e.e.* for (*R*) enantiomer in phosphate buffer 0.1 M pH 8, Table 1, Entry 1, Sub **3**) to acid one (30% *e.e.* for (*S*) enantiomer in MES buffer 1.2 M pH 5, Table 1, Entry 6, Sub **3**) along with a significant difference in the reaction rates (from 92% conversion to 20%).

This behavior could be explained in terms of both different electronic and steric properties of the imine substrates depending on the buffer solution, the pH and the ionic strength [47,48,49], in interaction with the different stabilized conformation of the [Ir(Cp*)(Van)Cl] complex [32,50].

Moreover, with the aim to evaluate the catalytic efficiency of the system here proposed, we determined the kinetic parameters and consequently its efficiency defined by *K*_cat_/*K*_M_ ratio [51,52,53] (see SI, Figure S6).

The affinity of Substrate **1**, 6,7-dimethoxy-1-methyl-3,4-dihydroisoquinoline was taken into consideration for comparison with other systems based on supramolecular interaction, reported in literature i.e., the systems based on the streptavidin/biotin technology. The kinetic parameters revealed that this catalyst could be considered an hybrid system with the substrate possessing a high affinity for the [Ir(Cp*)(Van)Cl] complex (*K*_M_ = 1.4 mM), while the catalytic activity and the overall efficiency (*K*_cat_ = 0.978 h^−1^, *K*_cat_/*K*_M_ = 0.688 h^−1^mM^−1^) is comparable to other artificial systems [54,55].

In conclusion, a hybrid catalyst generated from the interaction of the steric hindered and rigid vancomycin with Ir(III) metal complex was studied and characterized. The conformational rearrangement of vancomycin, caused by the complexation with IrCp*, was evinced by different analytical characterization techniques. The capability of this new hybrid catalyst to reduce different cyclic imines was evaluated in aqueous media under mild reaction conditions affording the reaction product with moderate to appreciable enantioselectivity. Particularly interesting were the results obtained in the asymmetric reduction of quinaldine **2** and of 3-methylbenzo[d]isothiazole 1,1-dioxide **3**. While for **2** an appreciable 61% *e.e.* was achieved in MES Buffer 1.2 M pH 5, for Substrate **3** a fascinating inversion of configuration occurred along with a good 42% *e.e.* by changing the buffer and its pH. This behavior might suggest the possibility for Van to create a different chiral coordination sphere around the catalytic center depending on the pH, ionic strength, and on the buffer, influencing the catalytic performance of the catalyst in function of the employed substrate. Indeed, the reproducibility of the in situ complexation mode was confirmed by the results obtained in independent experiments thus giving us a reasonable prospect for the possibility to improve the versatility of this system and the possibility to apply it to different types of enantioselective reactions, eventually changing the metal center to rhodium or ruthenium.

## 1. Experimental Section

In general, vancomycin was commercially available. [IrCp*Cl_2_]_2_ was synthetized as reported in the literature [56]. Catalytic reactions were monitored by HPLC analysis with a Merck–Hitachi L-7100 (Merck, Rome, Italy) equipped with a Detector UV6000LP (Thermo Fisher, Monza, Italy) and a chiral column (Chiralcel OD-H, Diacel, Tours, France). HR-MS analyses were performed by using a QTof Synapt G2 Si spectrometer (Waters, Milan, Italy) with an electrospray ionization source. The spectra were obtained by direct infusion of a sample solution in MeOH under ionization, ESI positive. ^1^H and ^13^C NMR spectra were recorded in D_2_O/[*d*_6_]DMSO (1% *v*/*v*) on Bruker Avance (Bruker, Milan, Italy) 600 MHz. Chemical shifts (δ) are expressed in parts per million (ppm). All the experiments were recorded at 298 K using TMS as the internal standard.

### 1.1. General Procedure for Synthesis of [Ir(Cp*)(Van)Cl] Complex

Van (1.1 eq, 25 mmM) was dissolved in 1 mL of water or the appropriate buffer. The dimer [IrCp*Cl_2_]_2_ (0.5 eq, 25 mM), opportunely dissolved in DMSO (10 µL), was added and the suspension was stirred for 1 h at room temperature. Then the yellow solution was used without further purification.

### 1.2. General Procedure for Asymmetric Transfer Hydrogenation.

[IrCp*Cl_2_]_2_ (1% mol) and Van (4% mol) were dissolved in 1 mL of MES buffer (MES 1.2 M, HCOONa 3 M, final pH 5) and stirred for 60 min at 25 °C. The substrate (final concentration 16 mM) was added to the catalyst solution. The reaction was stirred for 18 h at 25 °C. At the end of the reaction 10 µL of NaOH 10 N was added and the aqueous media was extracted with CH_2_Cl_2_ for Substrate **1** and **2** and with ethyl acetate for Substrate **3**. The organic layers were dried with anhydrous Na_2_SO_4_, filtered, and the solvent was removed under vacuum to be analyzed by HPLC equipped with a chiral column determining conversion and enantiomeric excess.

### 1.3. Circular Dichroism

The samples were prepared by diluting a stock solution of the [Ir(Cp*)(Van)Cl] complex (1 M water with 1% DMSO) to a final concentration of 2.5 mM in the appropriate buffer and sonicated for complete dissolution. Spectra were obtained from 280 to 450 nm with a 0.1 nm step and 1 s collection time per step, taking three averages. The spectrum of the buffer solution was subtracted to eliminate interference from cell, solvent, and optical equipment. The CD spectra were plotted as ellipticity θ (degree × cm^2^ × dmol^−1^) versus wavelength λ (nm). Noise-reduction was obtained using a Fourier-transform filter program from Jasco.

### 1.4. Kinetic Experiments

The stock solutions of the substrate were prepared in different concentrations (0.3–16 mM in DMF). Aliquots of 10 µl of the solution were mixture with 10 µL of stock solution of the [Ir(Cp*)(Van)Cl] complex (32 mM in DMF) in 1 mL buffer (MOPS 1.2 M, HCOONa 3 M, pH 5). After a suitable period of time (1–5 h), samples were collected and processed according to the experimental protocol. Conversions were determined by integration of the corresponding HPLC signals, using the calibration curve to correct for the difference in extinction coefficient for Substrate **1**. (Similar enantioselectivities were measured as in the experiments conducted under standard conditions). The curves for the saturation kinetics were determined in triplicate. Values obtained were plotted against the corresponding reaction times [57].

## Figures and Tables

**Figure 1:**
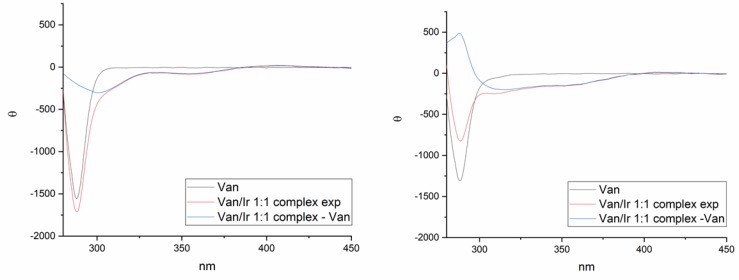
Circular dichroism (CD) spectra of free Vancomycin (Van) (black line) and Van/Ir 1:1 complex (red line) recorded in acetate buffer (pH 5, on the **left**) and in phosphate buffer (pH 8, on the **right**) are reported. The blue line is the obtained spectrum by subtracting Van contribution from the 1:1 complex. The concentration of Ir(III) is 2.5 mM.

**Figure 2:**
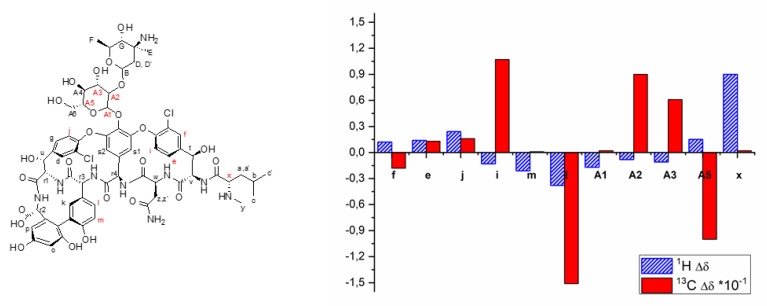
On the left: Van structure. On the right: Most significative chemical shift variations (ppm) of ^1^H and ^13^C resonances between free Van and the [Ir(Cp*)(Van)Cl] complex in the NMR spectra (see SI for complete assignment).

**Scheme 1:**
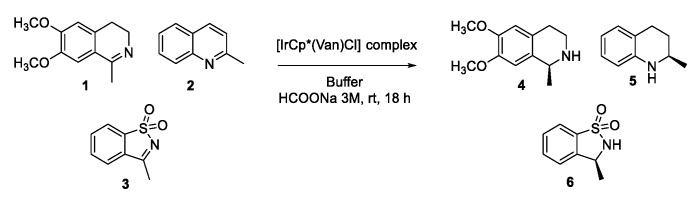
Screening of Asymmetric Transfer Hydrogenation (ATH) of cyclic imines.

**Table 1:** In situ ATH of different cyclic imines with the [Ir(Cp*)(Van)Cl] complex.

Entry	Buffer	Sub 1 Conv. % (*e.e.*%)	Sub 2 Conv. % (*e.e.*%)	Sub3 Conv. % (*e.e.*%)
1	Phosphate 0.1 M pH 8	56 (20, *R*) (a)	30 (36, *R*)	92 (42, *R*)
2	MOPS 1.2 M pH 7.8	34 (*rac*)	40 (46, *R*)	64 (*rac*)
3	MES 1.2 M pH 7	82 (4, *S*)	30 (9, *R*)	60 (4, *R*)
4	MES 1.2 M pH 6	40 (4, *S*)	67 (12, *R*)	25 (*rac*)
5	Acetate 0.1 M pH 5	34 (3, *S*)	20 (21, *R*)	30 (*rac*)
6	MES 1.2 M pH 5	75 (5, *S*)	35 (61, *R*)	20 (30, *S*)

Reaction conditions: substrate concentration 16 mM, 4 mol% Van, 1 mol% [IrCp*Cl_2_]_2_, buffer, HCOONa 3 M, 18 h and at 25 °C. (a) substrate 16 mM, 8 mol% Van, 1 mol% [IrCp*Cl_2_]_2_, buffer, HCOONa 3 M, 18 h and at 25 °C.

## Supplementary Materials

The following are available online at https://www.mdpi.com/1420-3049/24/15/2771/s1.
