# Supplementary material for: Vancomycin-Iridium (III) Interaction: An Unexplored Route for Enantioselective Imine Reduction
Source: Molecules. 2019 Jul 30;24(15):2771. doi: 10.3390/molecules24152771 (PMC6695689; doi:10.3390/molecules24152771)
Supplement: Supplementary file 1 [file molecules-24-02771-s001.pdf]

SUPPORTING INFORMATION

Vancomycin-iridium (III) interaction: an unexplored route for enantioselective imine reduction.

Giorgio Facchetti, Sara Pellegrino, Raffaella Bucci, Donatella Nava, Raffaella Gandolfi, Michael S. Christodoulou and Isabella Rimoldi

Dipartimento di Scienze Farmaceutiche, Università degli Studi di Milano, Via Golgi 19, 10033 Milano, Italia

*Corresponding author

e-mail: isabella.rimoldi@unimi.it.

Table of contents

1. Van/[IrCp*Cl$_2$]$_2$ complex characterization……………………..……………………………..……………..S3
1.1 HR-MS of $C_{76}H_{90}Cl_3IrN_9O_{24}$…………………………………………………………………………………S3
1.2 UV spectroscopy…………………………………………………………………...………………S4
1.3 NMR spectroscopy…………………………………………………………………………………S6
1.4 RAMAN spectrum………………………………………………………………………………...S18
1.5 Kinetic experiments…………………………………………………………………….……...S18
2. Additional catalysis data……………………………………………………..………..………S19
3. HPLC analysis………………………………………………………………….....…..…………..S20

Van/[IrCp*Cl$_2$]$_2$ complex characterization

1.1. MS of $C_{76}H_{90}Cl_2IrN_9O_{24}$

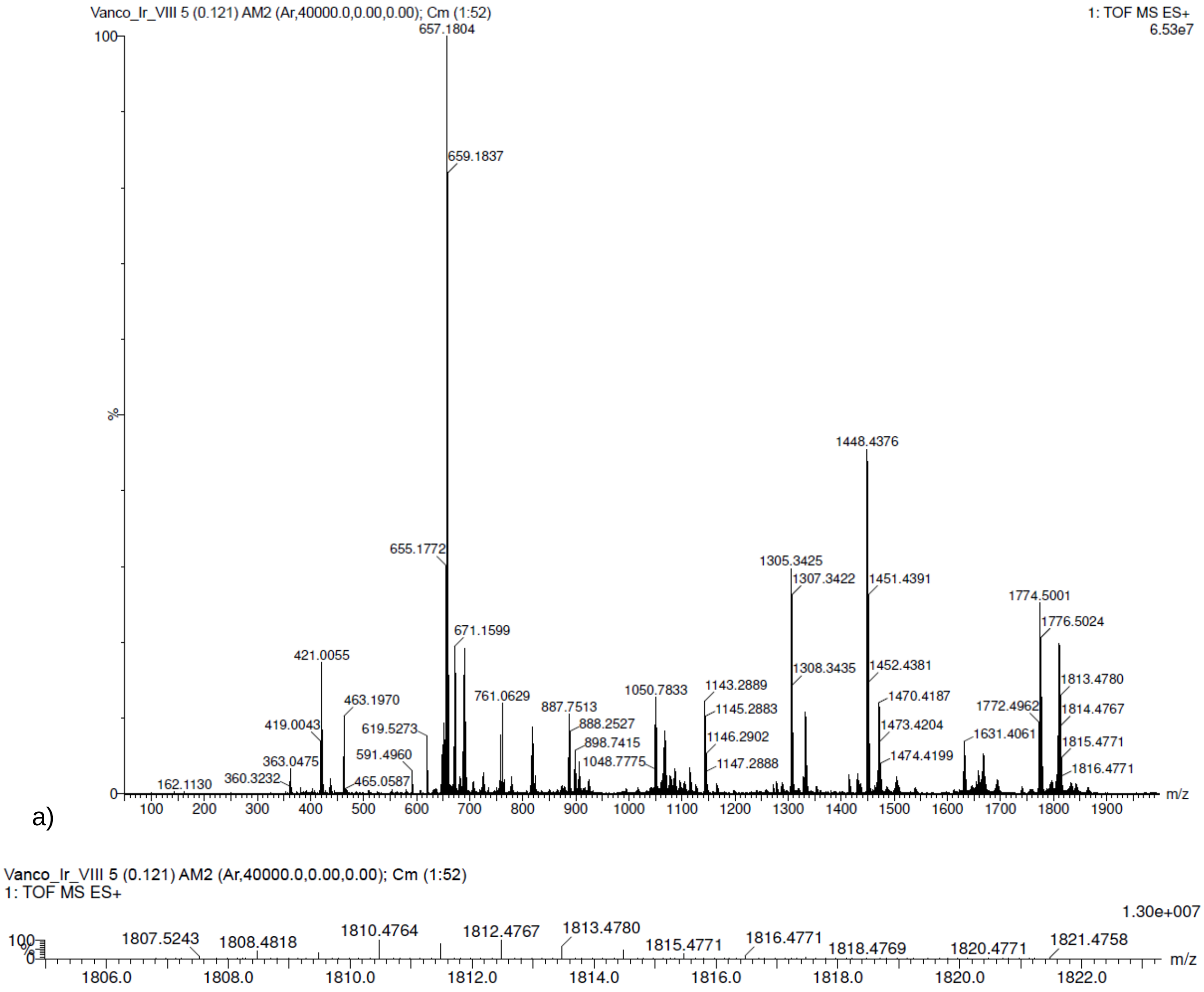

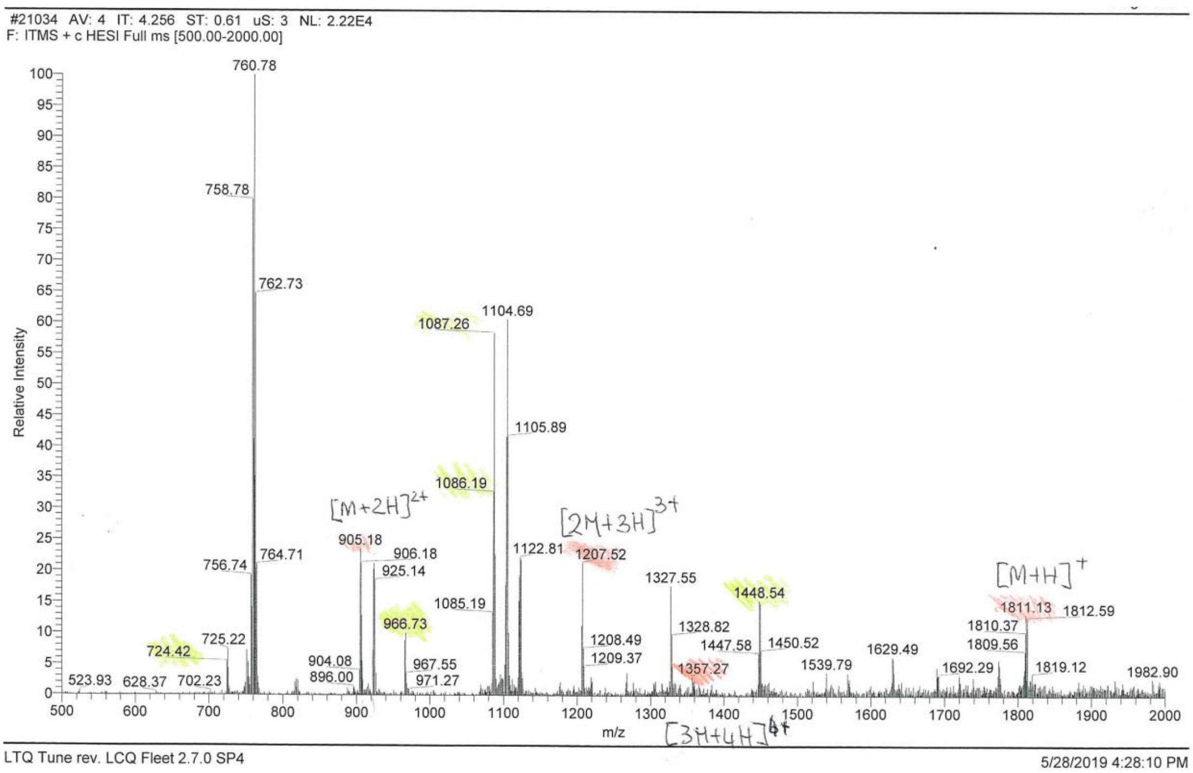

b)

Figure S1. a) MALDI-TOF and b) ESI spectra of [Ir(Cp*)(Van)Cl] complex (25 mM, 1:1 ratio Van/Ir) (red highlight for complex referred to free Van in green highlight).

1.2. UV spectroscopy

Stock solutions of [Ir(Cp*)(Van)Cl] complex (25 mM water with 1% DMSO) were diluted to a final concentration of 250 µM in the appropriate buffer and sonicated for complete dissolution.

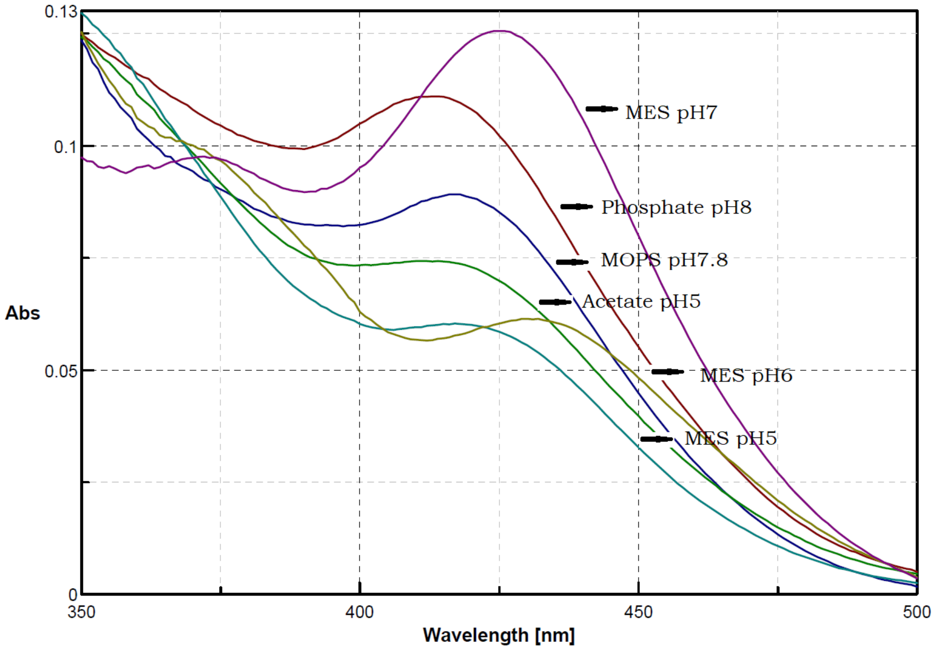

a)

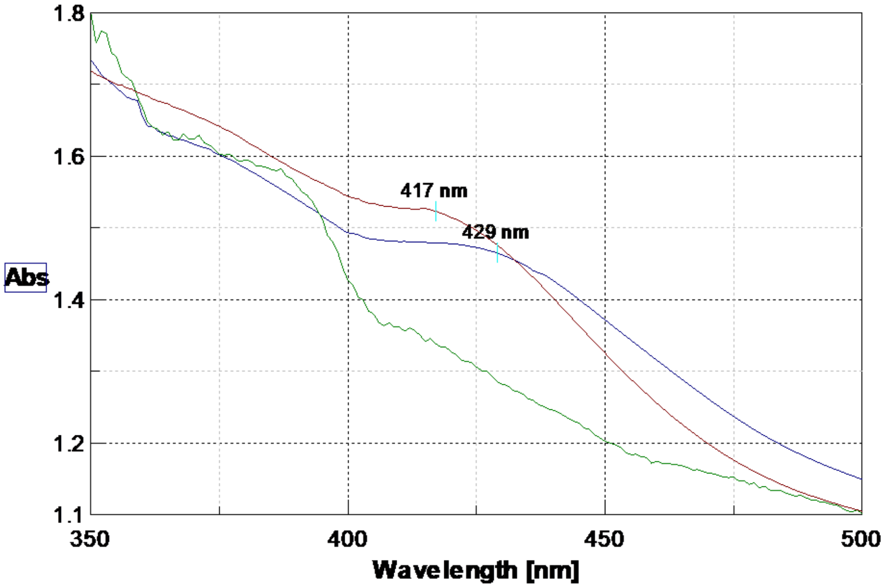

b)

Figure S2. a) UV spectra of Van/[IrCp*Cl2]2 complex (250 µM, 1:0.5 ratio) at different pH values; b) UV spectra of Van alone (green line), [IrCp*Cl2]2 alone (red line) and Van/[IrCp*Cl2]2 (blue line) in MES buffer pH 5.

1.3. **NMR spectroscopy**

Van was characterized by ^{1}H, ^{13}C, HSQC NMR experiments and [Ir(Cp*)(Van)Cl] complex by ^{1}H, ^{13}C, HSQC, TOCSY, ROESY, NOESY and COSY NMR experiments on 600 MHZ (^{1}H) and 150 MHZ (^{13}C) instruments. The spectra were recorded in D_2O with 1% [d_6]DMSO (25 mM, 298K). The proton naming convention is the one from Świątek et al. [1]

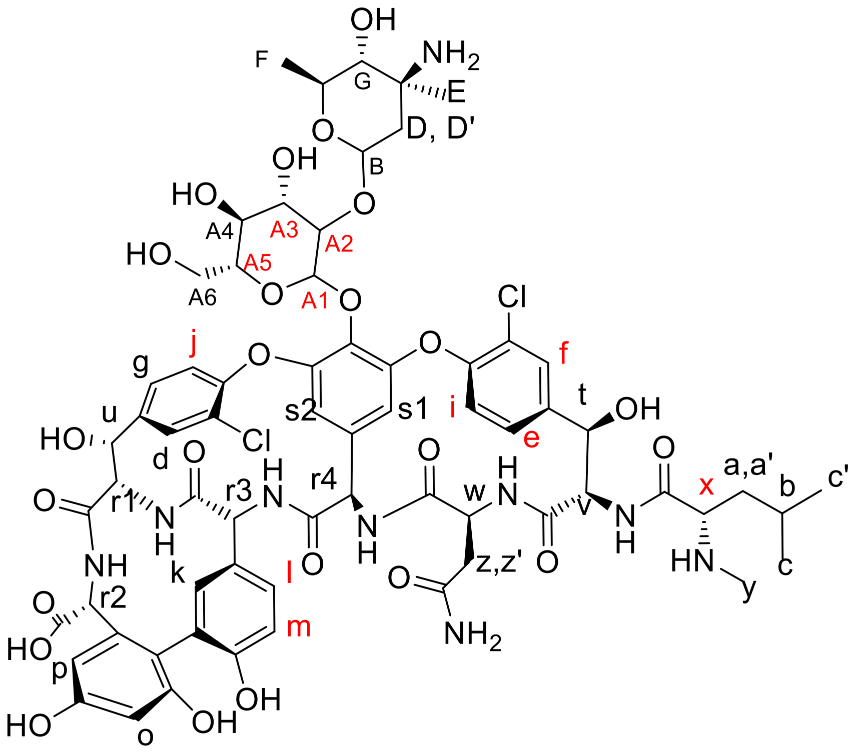

Table T1a: Vancomycin

Signal	^{1}H	^{13}C	Signal	^{1}H	^{13}C
d	7.67	128.3	r3	4.55	59.0
f	7.58	129.5^{a}	r2	4.21	63.1
e	7.52^{b}	126.4^{b}	w	4.08^{b}	60.6^{b}
g	7.52^{b}	126.4^{b}	r1	3.82	60.6^{b}
j	7.24^{b}	124.6^{b}	x	4.74^{b}	60.6^{b}
i	7.24^{b}	124.6^{b}	A2	3.74^{b}	60.6^{b}
k	7.11	135.6	A5	3.61^{b}	69.1^{b}
m	6.88	118.1^{b}	A6	3.73-4.04	60.7
l	6.87	118.1^{b}	A3	3.61^{b}	69.1^{b}
o	6.50	103.0	A4	3.61^{b}	69.1^{b}
p	6.44	107.8	G	3.41	70.7
r4	6.11	54.9	zz’	2.74-2.70	35.7
s1	5.74	-	y	2.72	31.8
u	5.53	71.4^{b}	DD’	2.03	33.0
A1	5.49	71.4^{b}	aa’	1.76-1.65	38.8
t	5.39	71.8	b	1.52	23.9
s2	5.38	105.4	E	1.40	22.3
B	5.31	97.8	F	1.12	16.2^{a}
v	4.84^{b}	64.0^{b}	cc’	0.81-0.78	21.8-21.6
c	4.84^{b}	64.0^{b}			

^{a}from HSQC analysis; ^{b}overlapped signals

Table T1b: [Ir(Cp*)(Van)Cl] complex

Signal	^{1}H	^{13}C	Signal	^{1}H	^{13}C
d	7.72	127.7[a,b]	r3	4.47	59.1
f	7.70	127.7[a,b]	r2	4.21	62.7[a]
e	7.67	127.7[a,b]	w	4.05[b]	60.8[a,b]
g	7.49[b]	126.2[b]	r1	3.84[b]	60.8[a,b]
j	7.48[b]	126.2[b]	x	3.85	60.8[a,b]
i	7.11[b]	135.3[b]	A2	3.68	69.1
k	7.11[b]	135.3[b]	A5	3.77	79.1
m	6.67	118.1	A3	3.50	76.2
l	6.49[b]	103.0[b]	A6	3.84-4.04	60.6[a]
o	6.49[b]	103.0b	A4	3.69	69.1
p	6.41	107.8	G	3.41	70.7
r4	6.17	55.2	zz’	2.77-2.73	35.7
s1	5.66	101.0[a]	y	2.73	32.0
u	5.52	72.1	DD’	2.01	33.0
A1	5.32	71.6[b]	aa’	1.78-1.61	38.7
t	5.29	71.6[b]	b	1.46	24.0
s2	5.37	107.5[a]	E	1.44	22.2
B	5.35	97.6	F	1.06	13.1
v	4.80	64.0[b]	cc’	0.80-0.75	21.7
c	4.76	64.0[b]			

[a]from HSQC analysis; [b]overlapped signals

Table T1c: Shift difference analysis between Van and [Ir(Cp*)(Van)Cl] complex.

Signal	^{1}H	^{13}C	Signal	^{1}H	^{13}C
d	+0.05	-0.6	r3	-0.08	+0.1
f	+0.12	-1.8	r2	-	-0.05
e	+0.15	+1.3	w	-0.03	+0.2
g	-0.03	-0.2	r1	+0.02	+0.2
j	+0.24	+1.6	x	-0.89	+0.2
i	-0.13	+10.7	A2	-0.08	+8.5
k	-	-0.3	A5	+0.15	-10
m	-0.21	-	A6	-	-0.1
l	-0.38	-15.1	A3	-0.11	+6
o	-0.01	-	A4	+0.08	-
p	-0.03	-	G	-	-
r4	+0.06	+0.3	zz’	-	-
s1	-0.08	-	y	+0.01	+0.2
u	-0.01	+0.7	DD’	-0.02	-
A1	-0.17	+0.2	aa’	0.02-0.04	-0.1
t	-0.1	-0.2	b	-0.06	+0.1
s2	-0.01	+2.1	E	+0.04	-0.1
B	+0.04	-0.2	F	-0.06	-2.9
v	-0.04	-	cc’	0.01-0.03	-
c	-0.08	-			

Figure S3. ^{1}H, ^{13}C-NMR and HSQC of Van.

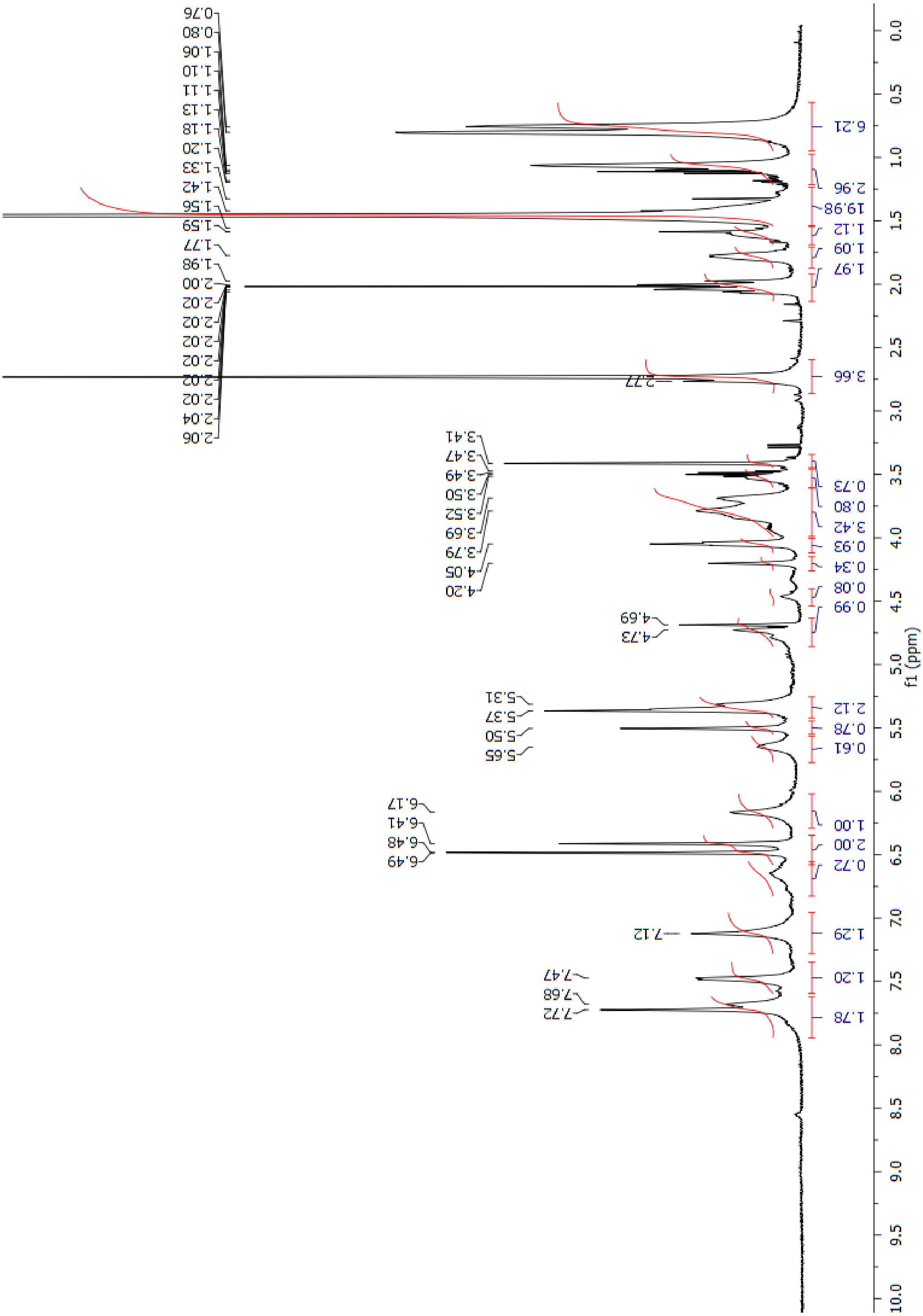

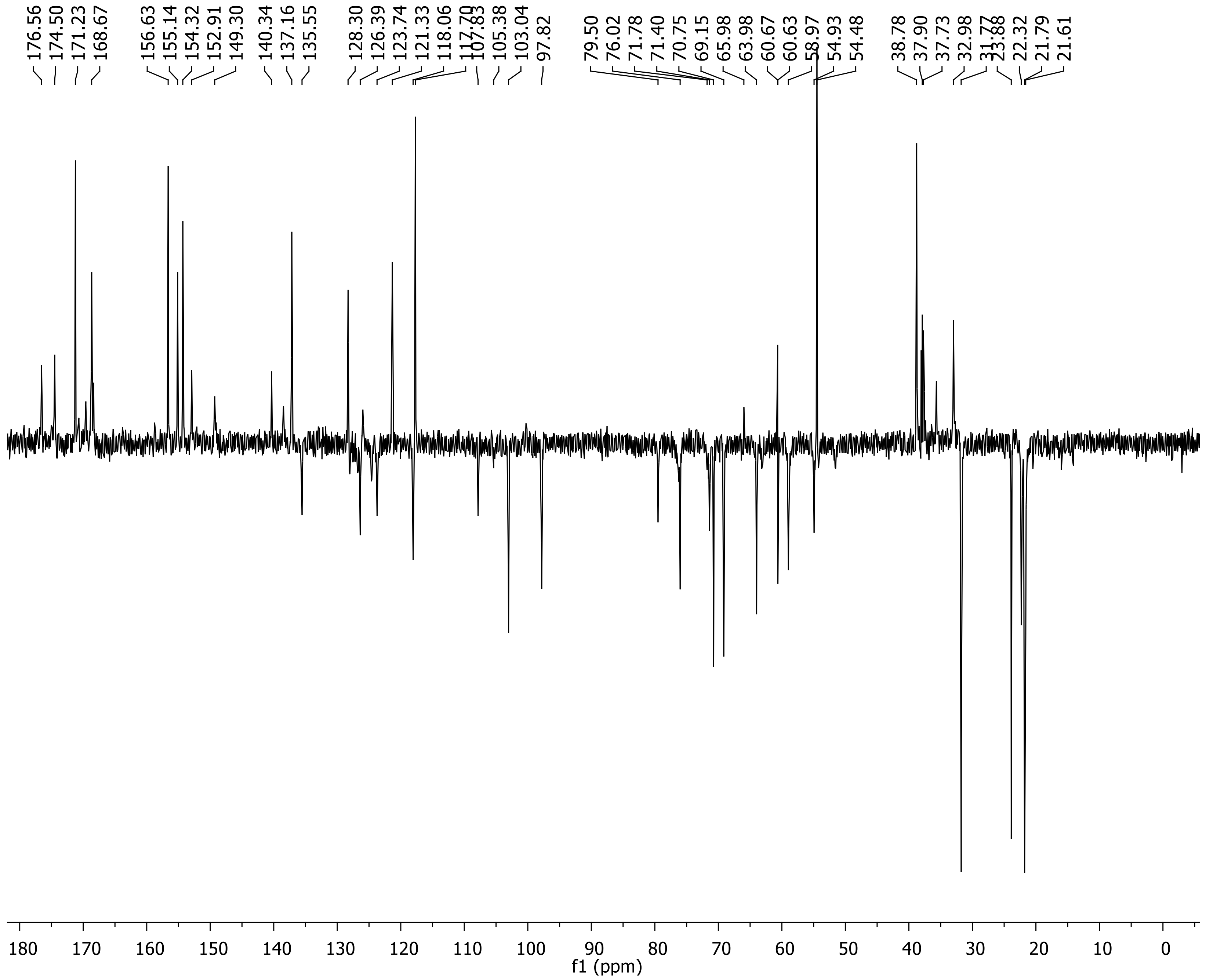
176.56
174.50
171.23
168.67
156.63
155.14
154.32
152.91
149.30
140.34
137.16
135.55
128.30
126.39
123.74
121.33
118.06
117.70
107.83
105.38
103.04
97.82
79.50
76.02
71.78
71.40
70.75
69.15
65.98
63.98
60.67
60.63
58.97
54.93
54.48
38.78
37.90
37.73
32.98
31.77
23.88
22.32
21.79
21.61
f1 (ppm)

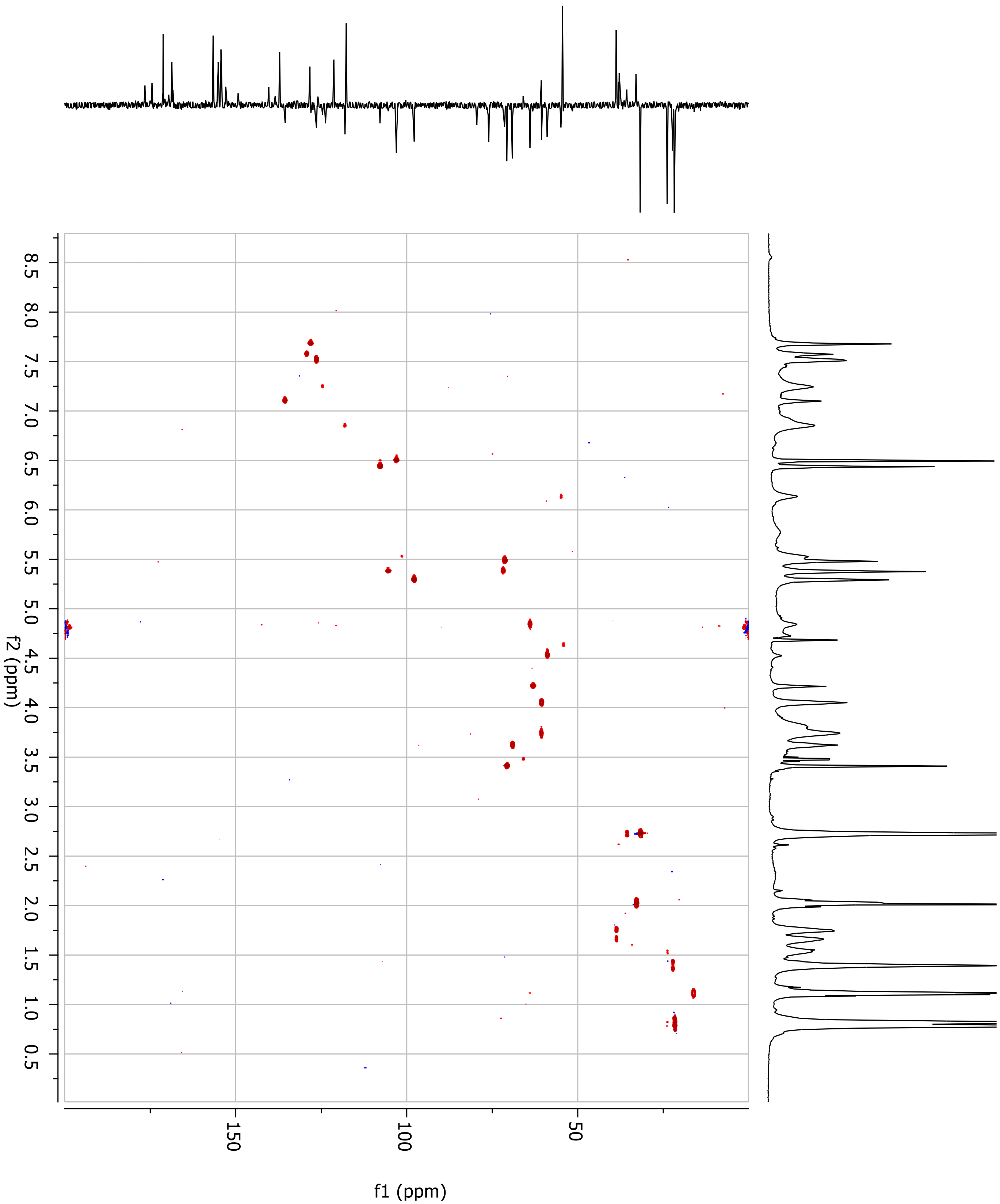
f2 (ppm)
f1 (ppm)

Figure S4. ^{1}H, ^{13}C-NMR, HSQC, TOCSY, ROESY, NOESY and COSY of [Ir(Cp*)(Van)Cl] complex.

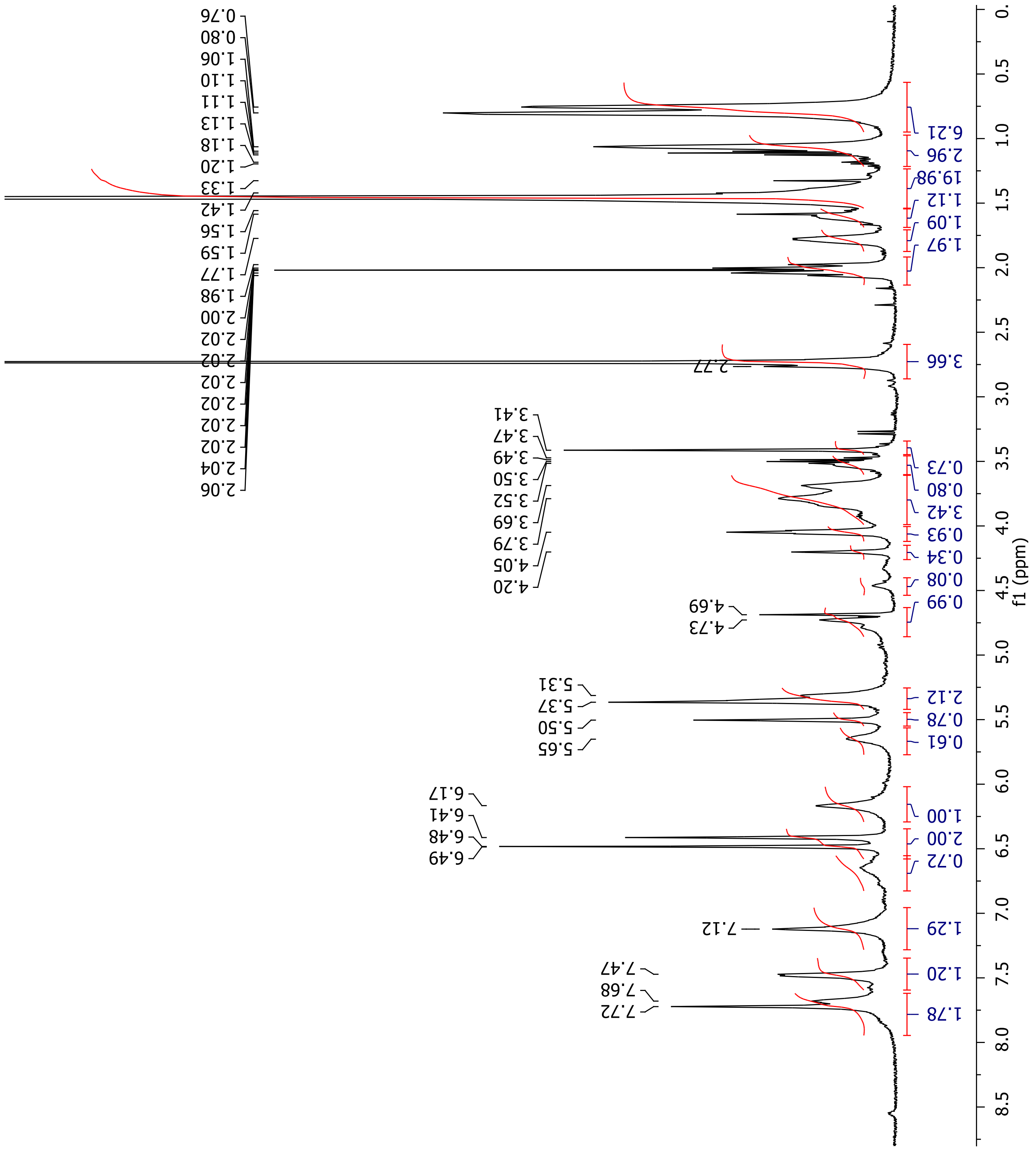

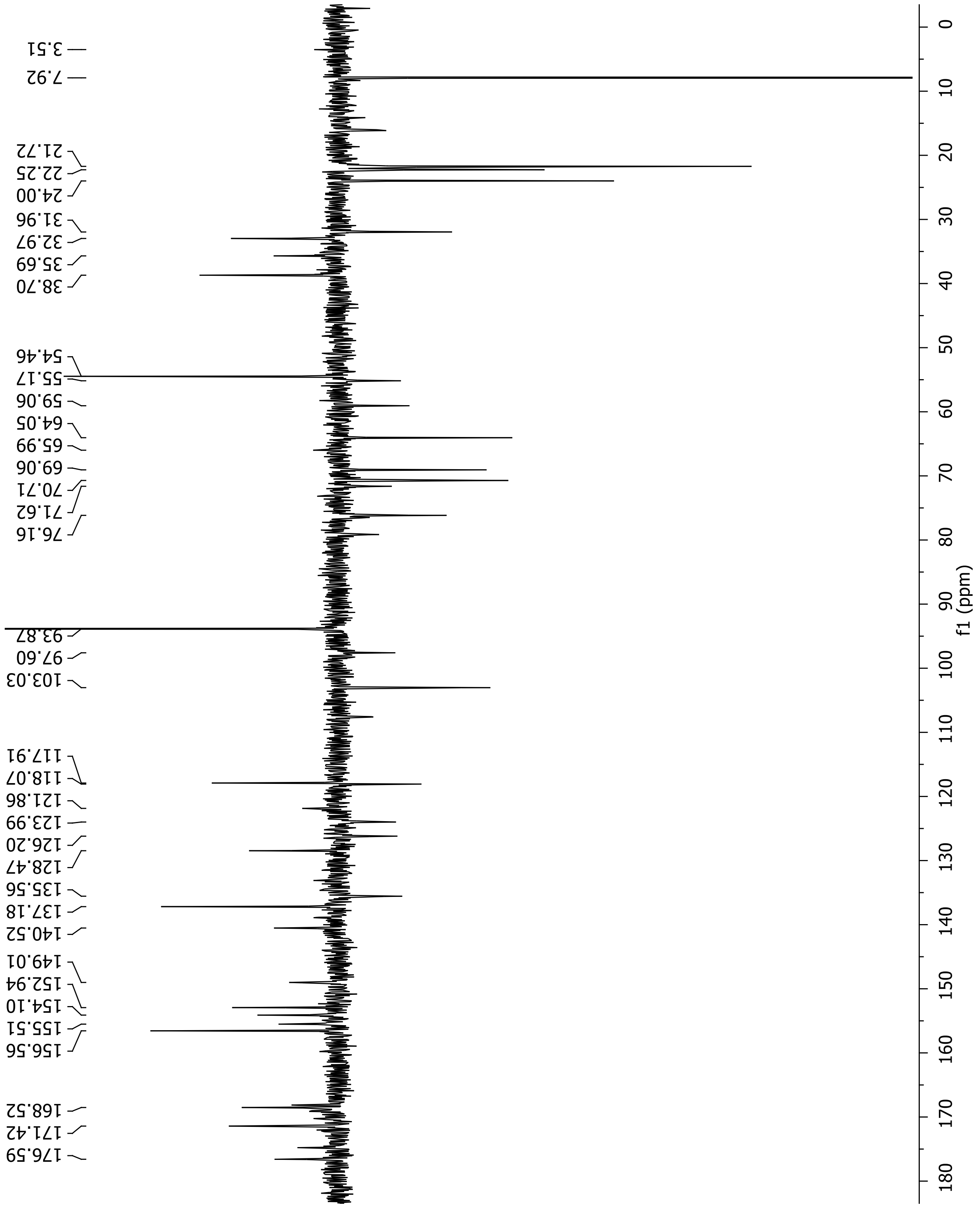
3.51
7.92
21.72
22.25
24.00
31.96
32.97
35.69
38.70
54.46
55.17
59.06
64.05
65.99
69.06
70.71
71.62
76.16
93.87
97.60
103.03
117.91
118.07
121.86
123.99
126.20
128.47
135.56
137.18
140.52
149.01
152.94
154.10
155.51
156.56
168.52
171.42
176.59
f1 (ppm)

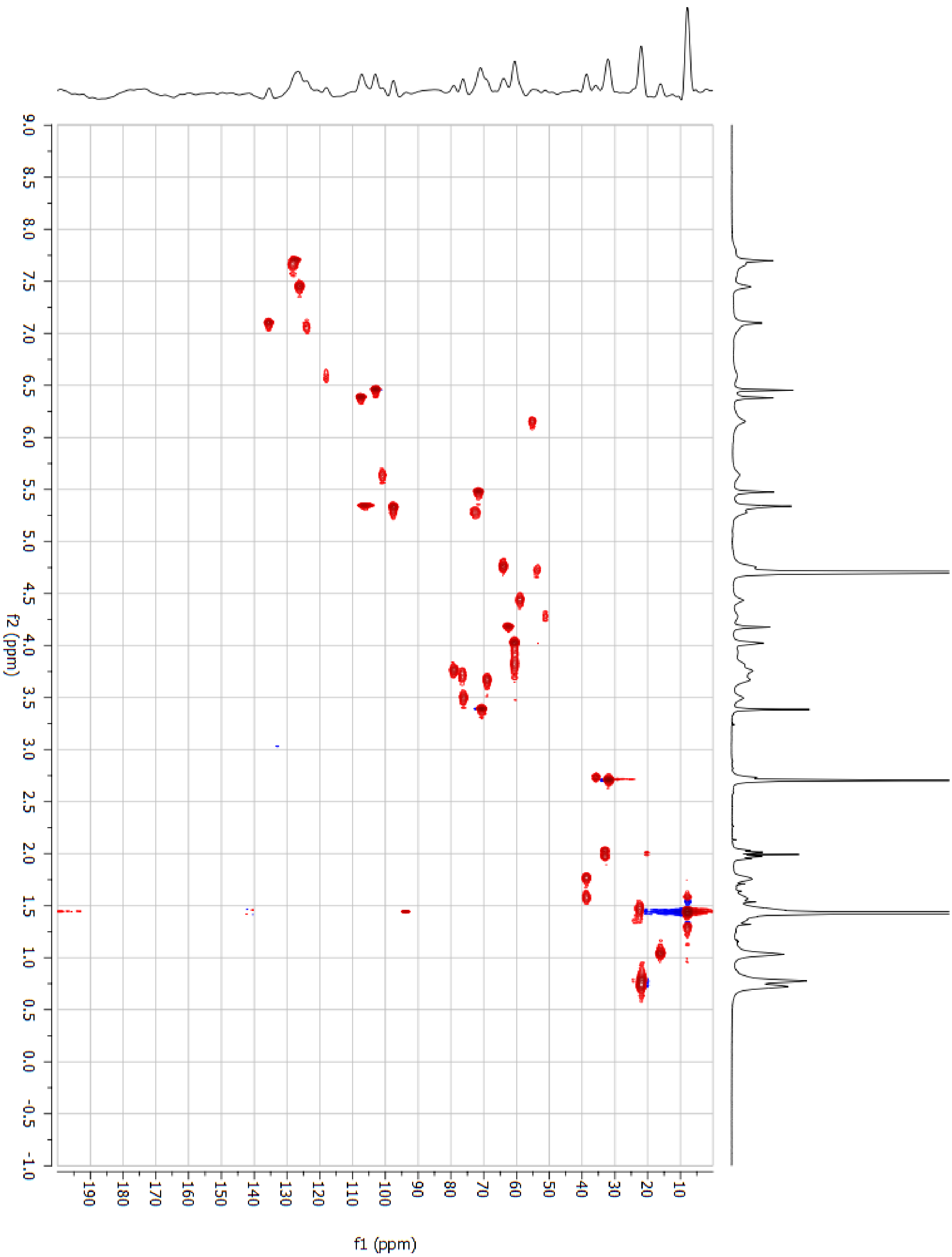

HSQC of [Ir(Cp*)(Van)Cl] complex

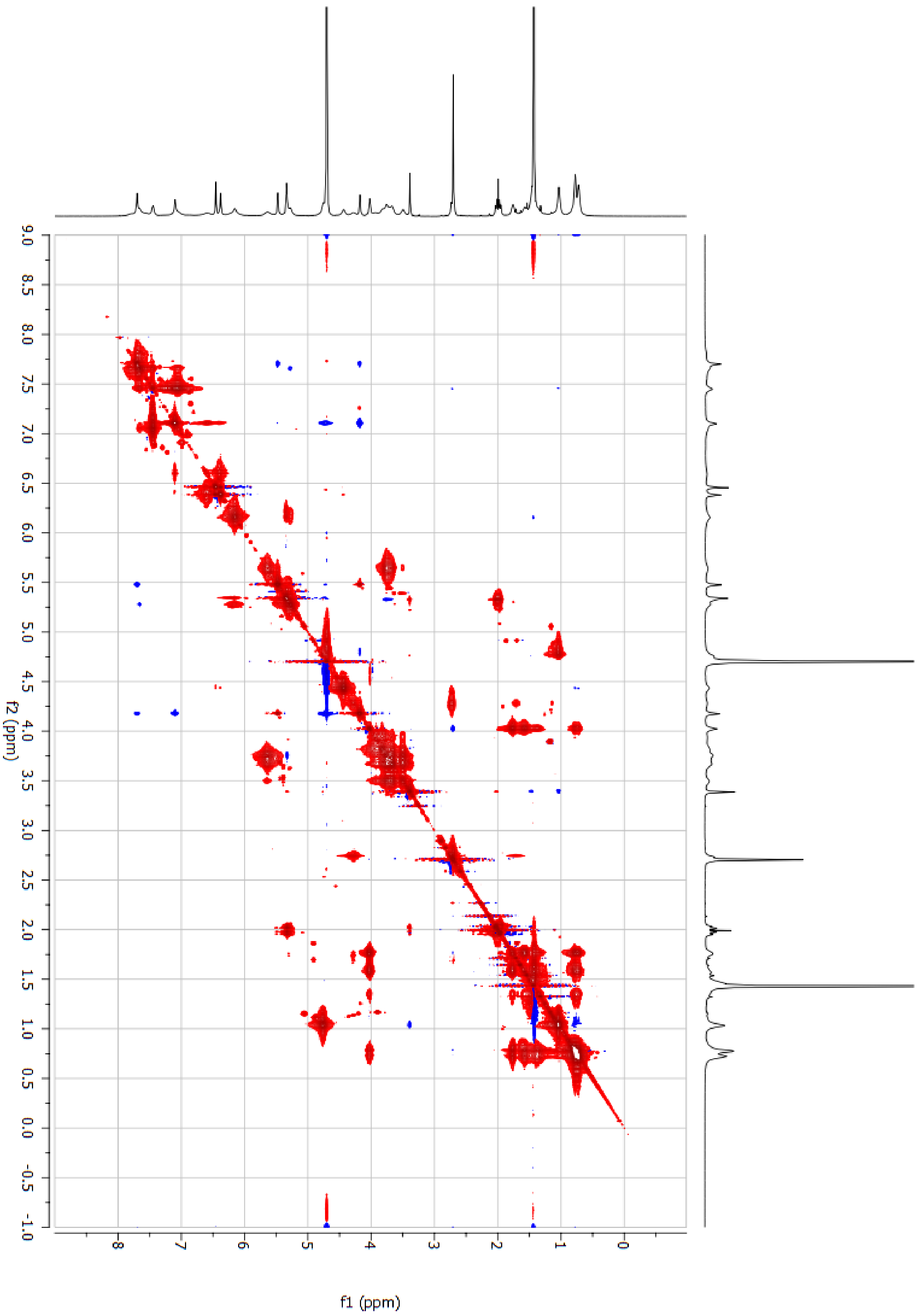

TOCSY of [Ir(Cp*)(Van)Cl] complex

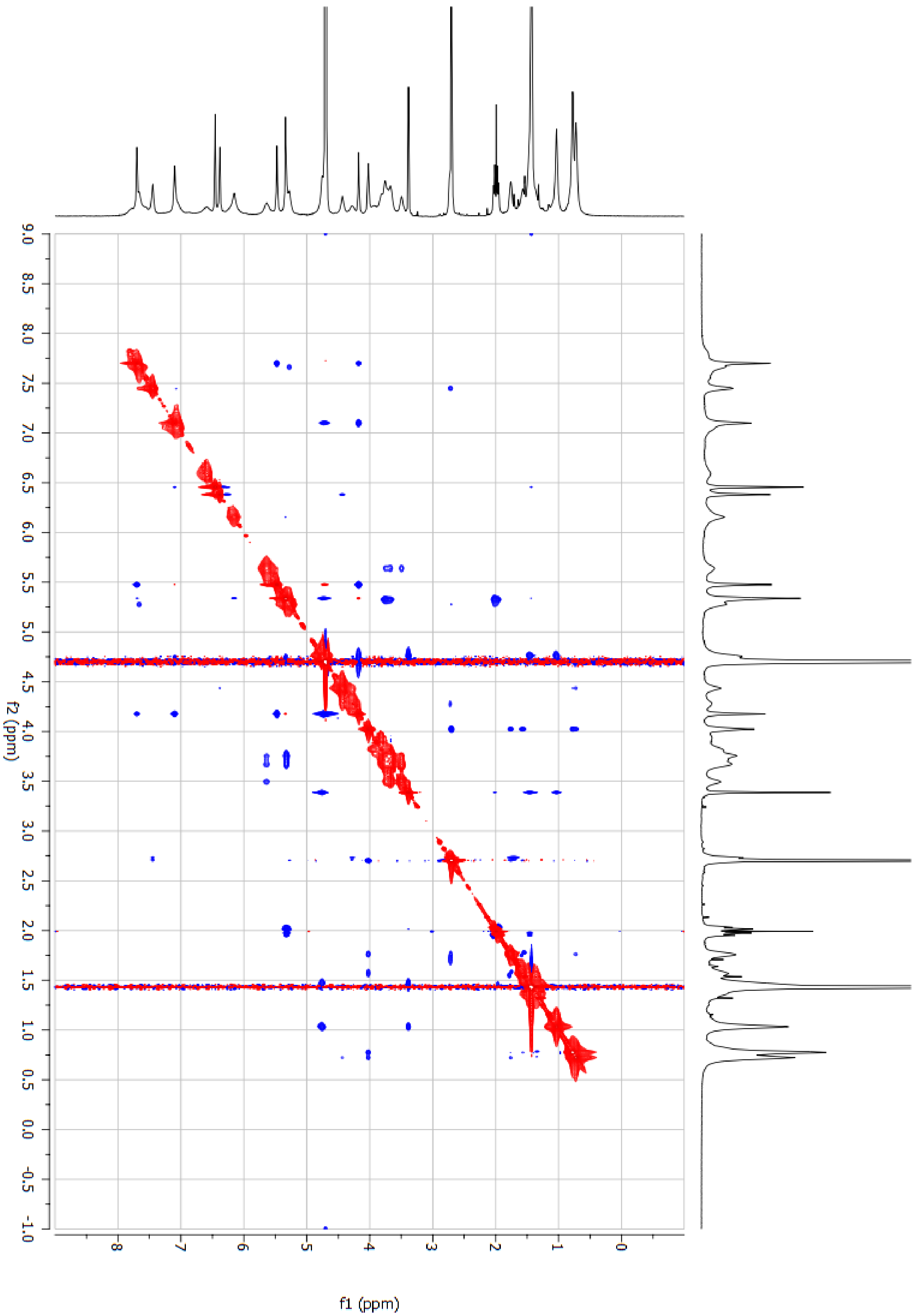

ROESY of [Ir(Cp*)(Van)Cl] complex

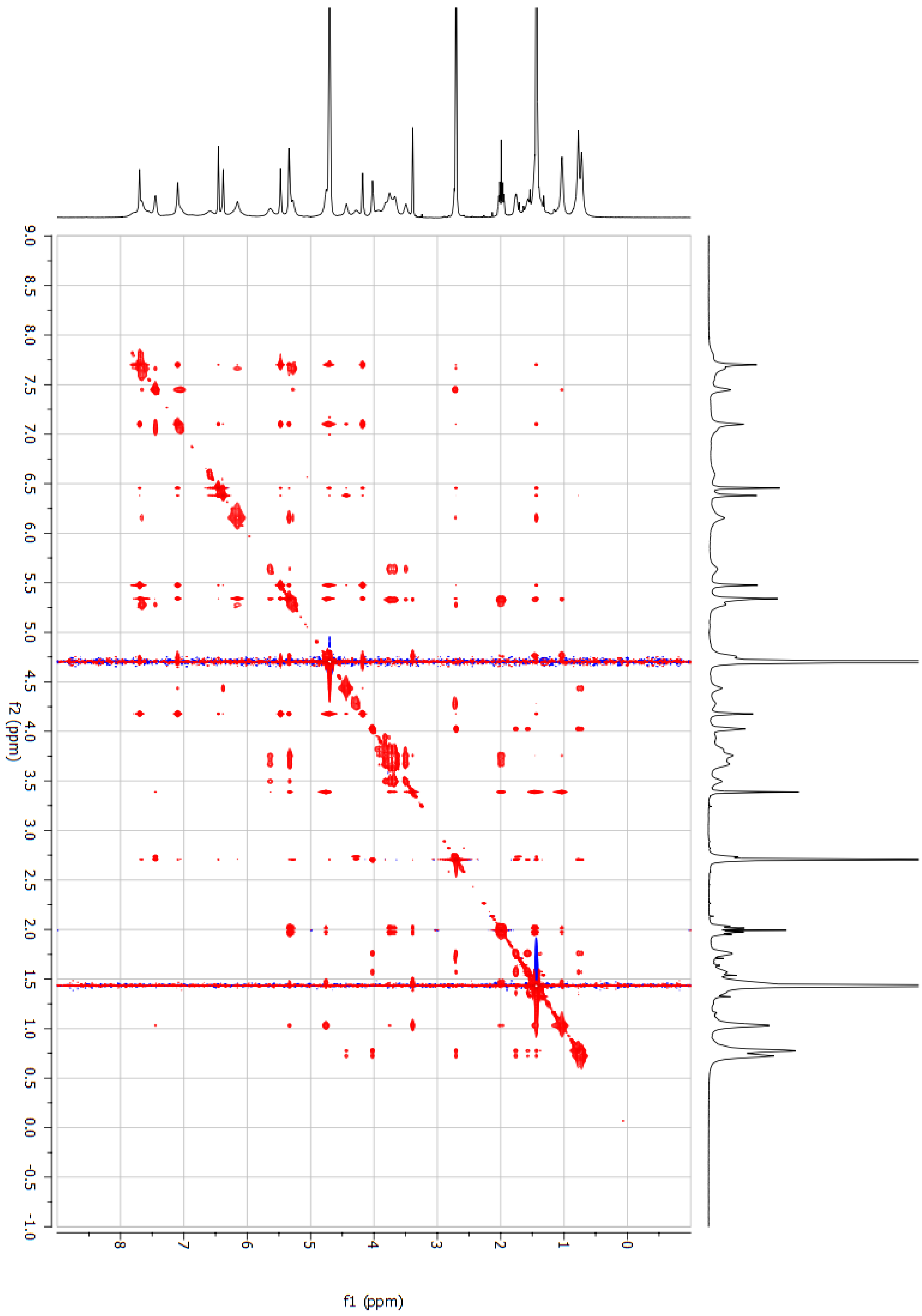

NOESY of [Ir(Cp*)(Van)Cl] complex

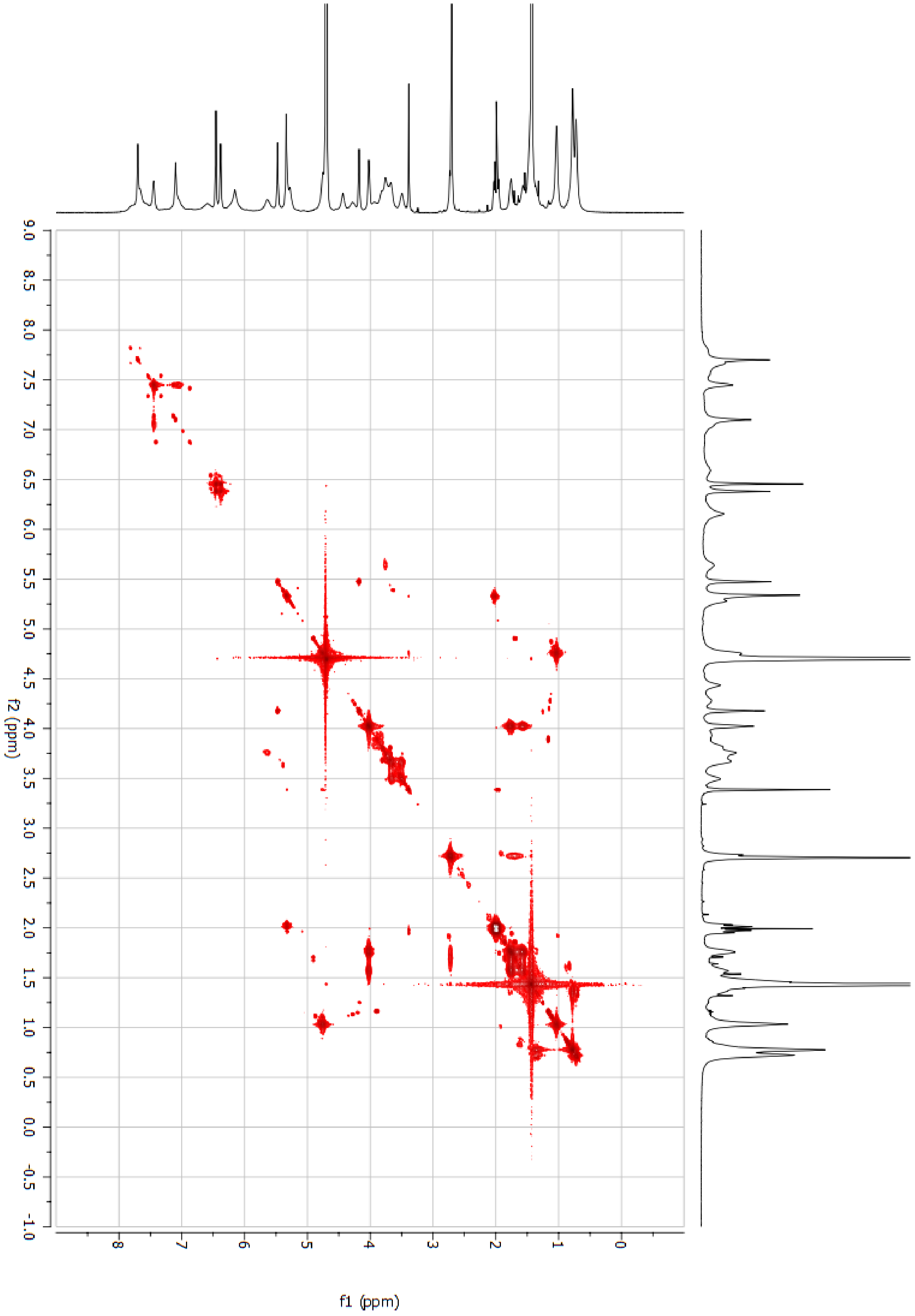

COSY of [Ir(Cp*)(Van)Cl] complex

1.4. **Raman Spectroscopy**

The Raman spectra were obtained using a diode laser with a 1064 nm excitation wavelength and an 80-mW output power directed towards a sample holder with the cuvette for sample irradiation.

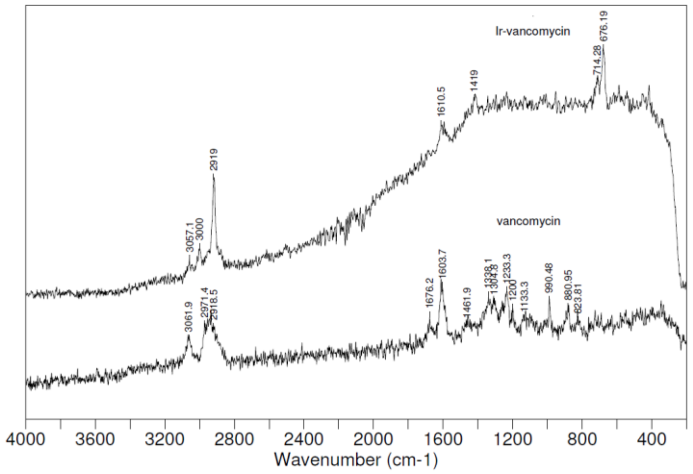

Figure S5. Raman spectra for overlay of 25 mM vancomycin alone (the band at 1603 cm^{-1}, attributed to carbonyl group; the band at 1338 cm^{-1} attributed to CH_3 bending, the band at 990 cm^{-1} represents breathing of the aromatic ring and the band at 880 cm^{-1} represents the stretching of the C-C bond) and of 25 mM [Ir(Cp*)(Van)Cl] complex.[2]

1.5. **Kinetic experiments**

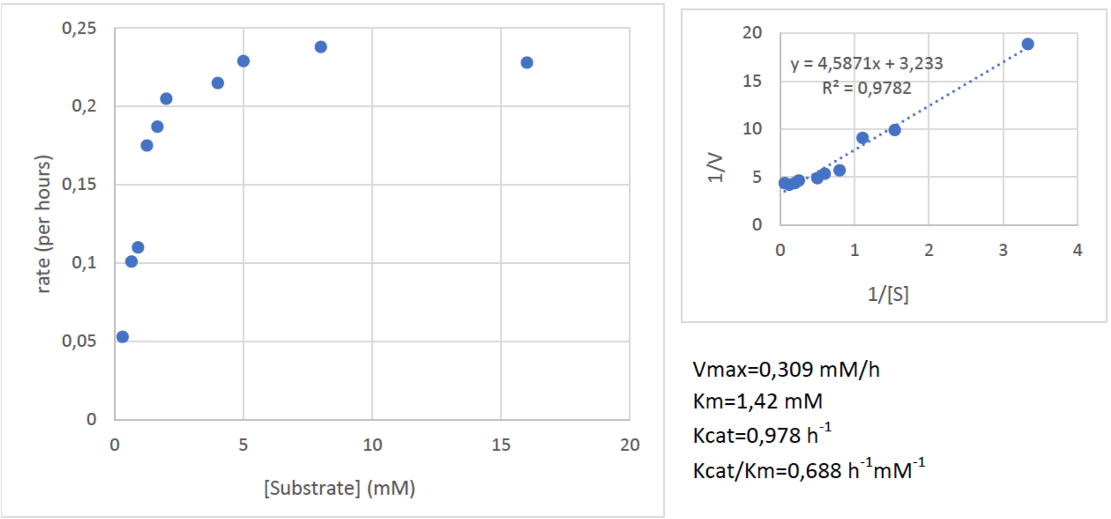

Figure S6: Kinetic parameter describing the reduction of 6,7-dimethoxy-1-methyl-3,4-dihydroisoquinoline **1** by [Ir(Cp*)(Van)Cl] complex in ATH reaction conditions.

2. Additional catalysis data

Table T2. Evaluation of different reaction conditions for ATH of cyclic imines

Entry	Buffer	Van/Ir ratio	$[sub]_{final}$ mM	Temp	**1** conv.%(e.e.%)	**2** conv.%(e.e.%)	**3** conv.%(e.e.%)
1	Phosphate 0.1 M pH 8	0:1	16	25°C	32	-	63
2	MOPS 1.2 M pH 7.8	0:1	16	25°C	38	-	61
3	MES 1.2 M pH 7	0:1	16	25°C	85	4	44
4	MES 1.2 M pH 6	0:1	16	25°C	99	8	35
5	Acetate 0.1 M pH 5	0:1	16	25°C	99	10	18
6	MES 1.2 M pH 5	0:1	16	25°C	99	12	13
7	Phosphate 0.1 M pH 8	1:0	16	25°C	-	-	-
8	MOPS 1.2 M pH 7.8	1:0	16	25°C	-	-	-
9	MES 1.2 M pH 7	1:0	16	25°C	-	-	-
10	MES 1.2 M pH 6	1:0	16	25°C	-	-	-
11	Acetate 0.1 M pH 5	1:0	16	25°C	-	-	-
12	Phosphate 0.1 M pH 8	2:1	16	25°C	42 (8 *S*)	30 (36 *R*)	92 (42 *R*)
13	MOPS 1.2 M pH 7.8	2:1	16	25°C	34 (*rac*)	40 (46 *R*)	64 (*rac*)
14	MES 1.2 M pH 7	2:1	16	25°C	82 (4 *S*)	30 (9 *R*)	60 (4 *R*)
15	MES 1.2 M pH 6	2:1	16	25°C	40 (4 *S*)	67 (12 *R*)	25 (*rac*)
16	Acetate 0.1 M pH 5	2:1	16	25°C	34 (*rac*)	20 (21 *R*)	30 (*rac*)
17	MES 1.2 M pH 5	2:1	16	25°C	75 (*rac*)	35 (61 *R*)	20 (30 *S*)
18	MES 1.2 M pH 5	1:1	16	25°C	63 (*rac*)	31 (22 *R*)	21 (28 *S*)
19	MES 1.2 M pH 5	2:1	32	25°C	25 (*rac*)	15 (23 *R*)	8 (4 *R*)
20	MES 1.2 M pH 5	2:1	16	25°C	60 (*rac*)	40 (29 *R*)	39 (23 *S*)
21	MES 1.2 M pH 5	2:1	32	25°C	31 (*rac*)	18 (22 *R*)	15 (29 *S*)
22	MES 1.2 M pH 5	2:1	16	25°C	-	8 (15 *R*)	16 (30 *S*)
23	MES 1.2 M pH 5	2:1	16	10°C	-	13 (43 *R*)	n.d.
24	MES 1.2 M pH 5	2:1	16	40°C	95 (*rac*)	50 (26 *R*)	36 (16 *S*)
25	Phosphate 0.1 M pH 8	4:1	16	25°C	56 (20 *S*)	20 (10 *R*)	70 (35 *R*)
26	MOPS 1.2 M pH 7.8	4:1	16	25°C	49 (*rac*)	20 (*rac*)	70 (11 *R*)
27	MES 1.2 M pH 7	4:1	16	25°C	87 (5 *S*)	19 (50 *R*)	62 (5 *R*)
28	MES 1.2 M pH 6	4:1	16	25°C	55 (*rac*)	30 (17 *R*)	22 (*rac*)
29	Acetate 0.1 M pH 5	4:1	16	25°C	38 (4 *S*)	20 (37 *R*)	21 (*rac*)
30	MES 1.2 M pH 5	4:1	16	25°C	4 (*rac*)	23 (48 *R*)	18 (38 *S*)

31	MES<br>1.2 M pH 5	4:1	16	10°C	-	35 (47 *R*)	n.d.

Reaction conditions: HCOONa 3 M, 18 h, activation time 60 min.

3. HPLC analysis

Substrate **1**: eluent hexane/ethanol/DEA=95/5/0.1; λ=283 nm; flow=1.0 mL/min; retention time for **1** 10.9 min; enantiomers of **4**: t_S=15.2 min; t_R=18.7 min.

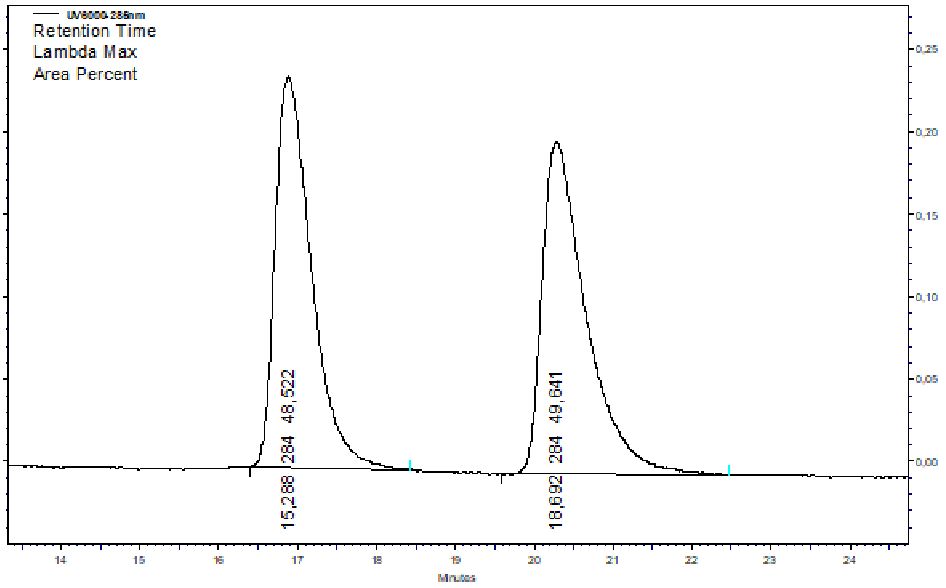

Figure S7. HPLC of standard for (*R*) and (*S*)- 6,7-dimethoxy-1-methyl-1,2,3,4-tetrahydroisoquinoline.

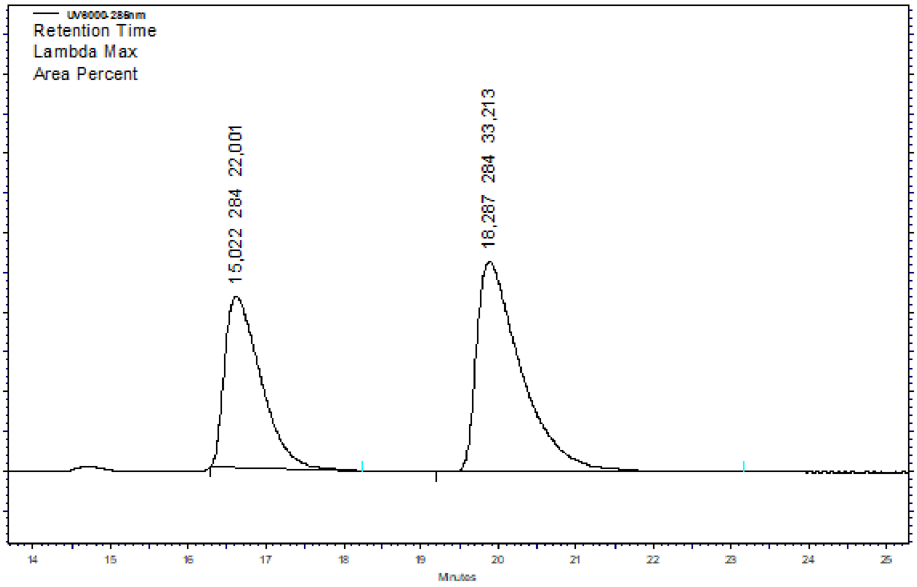

Figure S8. ATH of **1** in optimized reaction conditions (see manuscript, Table 1, entry 1-sub **1**).

Substrate **2**: eluent hexane/*iso*-propanol=90/10; λ=254 nm; flow=0.8 mL/min; retention time for **2** 14.8 min; enantiomers of **5**: t_S=6.6 min; t_R=7.4 min.

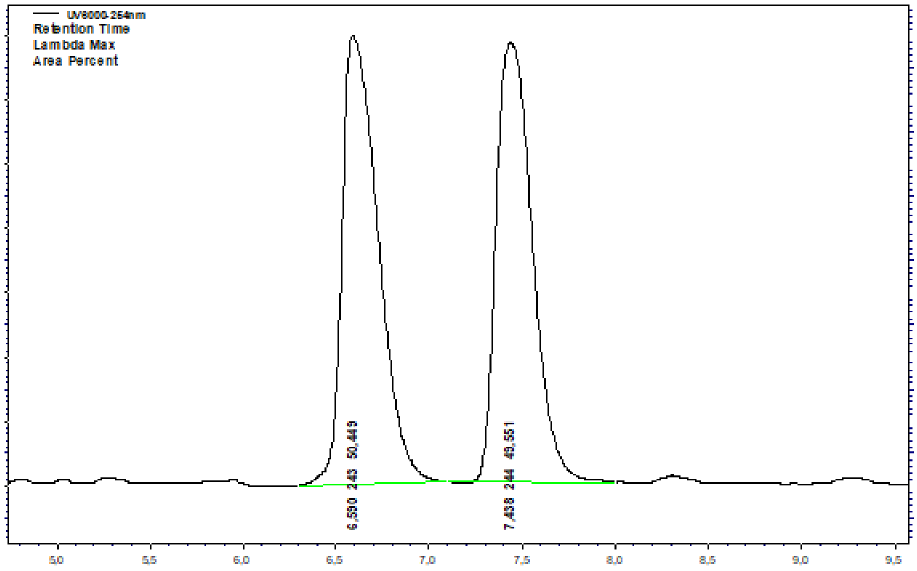

Figure S9. HPLC of standard for (*R*) and (*S*)- 2-methyl-1,2,3,4-tetrahydroquinoline.

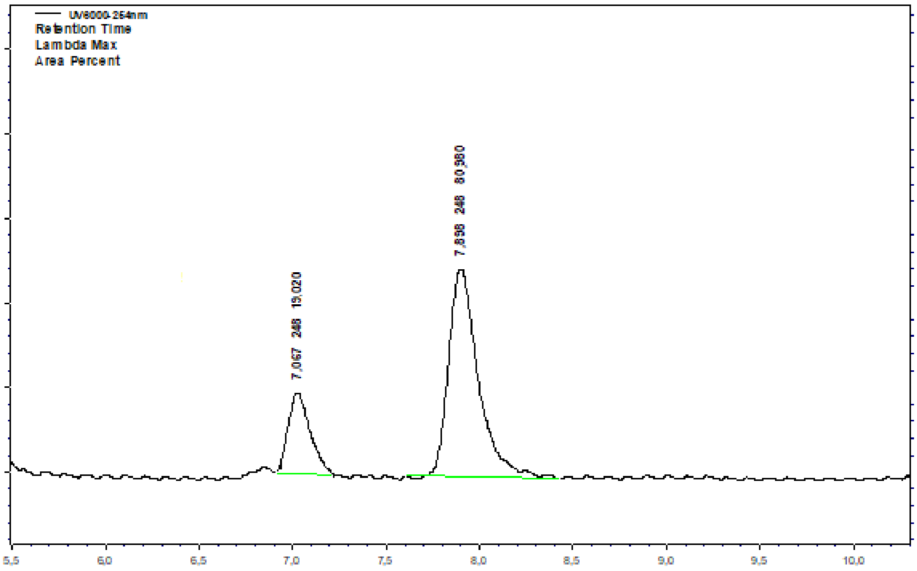

Figure S10. ATH of **2** in optimized reaction conditions (see manuscript, Table 1, entry 6-sub **2**).

Substrate **3**: eluent hexane/*iso*-propanol=80/20; λ=220 nm; flow=0.7 mL/min; retention time for **3** 28.6 min; enantiomers of **6**: $t_{(S)}$=19.5 min; $t_{(R)}$=24.6 min.[3]

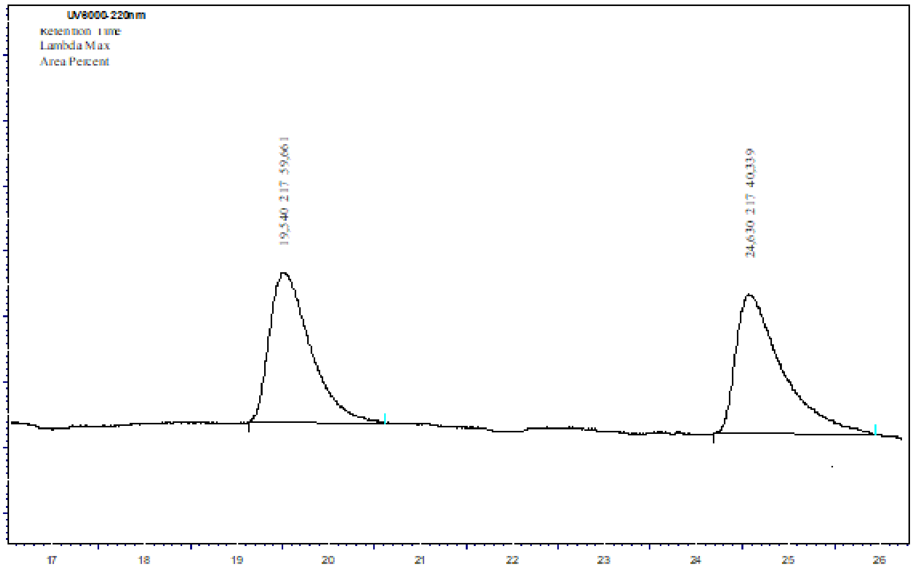

Figure S11. HPLC of standard for (*R*) and (*S*)- 3-methyl-2,3-dihydrobenzo[d]isothiazole 1,1-dioxide.

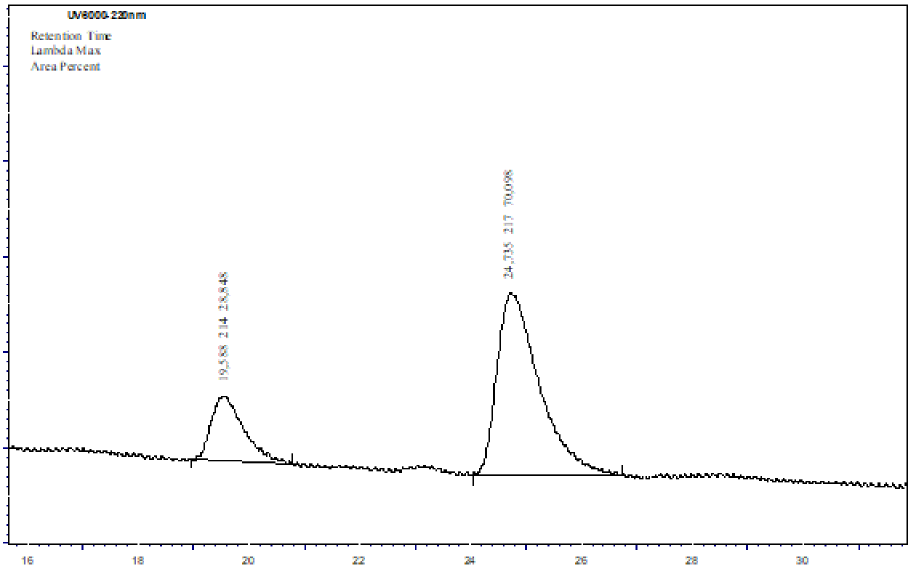

Figure S12. ATH of **3** under optimized reaction conditions (see manuscript, Table 1, entry 1-sub **3**).

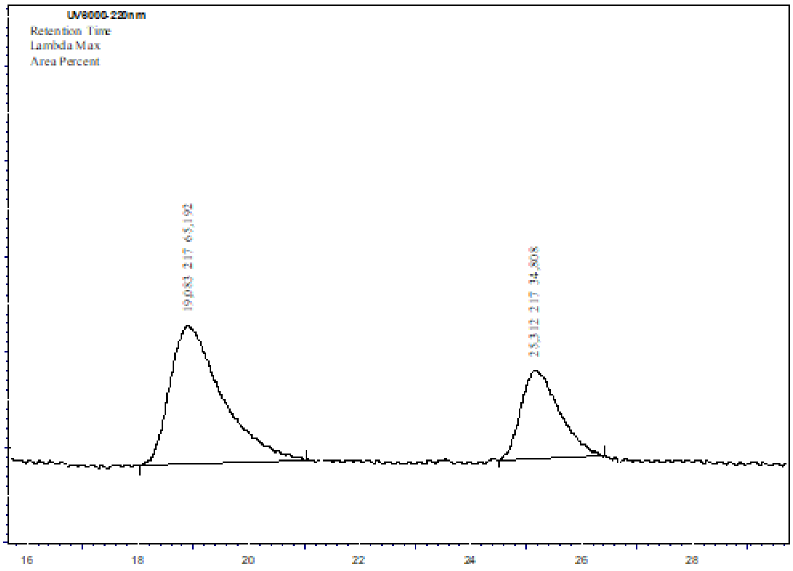

Figure S13. ATH of **C** under optimized reaction conditions (see manuscript, Table 1, entry 6-sub **3**).

REFERENCES

[1] a) M. Świątek, D. Valensin, C. Migliorini, E. Gaggelli, G. Valensin, M. Jeżowska-Bojczuk, *Dalton Trans.* **2005**, 3808-3813; b) J. Treviño, C. Bayón, A. Ardá, F. Marinelli, R. Gandolfi, F. Molinari, J. Jimenez-Barbero, M. J. Hernáiz, *Chem. Eur. J.* **2014**, *20*, 7363-7372; c) C. M. Pearce, D. H. Williams, *Journal of the Chemical Society, Perkin Transactions 2* **1995**, 153-157.

[2] a) R. C. Lora, L. Silveira, S. R. Zamuner, M. T. T. Pacheco, *Spectroscopy* **2011**, *25*; b) P. S. Nejman, B. Morton-Fernandez, D. J. Moulding, K. S. Athukorala Arachchige, D. B. Cordes, A. M. Z. Slawin, P. Kilian, J. D. Woollins, *Dalton Trans.* **2015**, *44*, 16758-16766.

[3] Y.-Q. Wang, S.-M. Lu, Y.-G. Zhou, *J. Org. Chem.* **2007**, *72*, 3729-3734.
